# Supplementary material for: TCO, a Putative Transcriptional Regulator in Arabidopsis, Is a Target of the Protein Kinase CK2
Source: Int J Mol Sci. 2018 Dec 28;20(1):99. doi: 10.3390/ijms20010099 (PMC6337506; doi:10.3390/ijms20010099)
Supplement: Supplementary file 1 [file ijms-20-00099-s001.pdf]

Supplementary Data

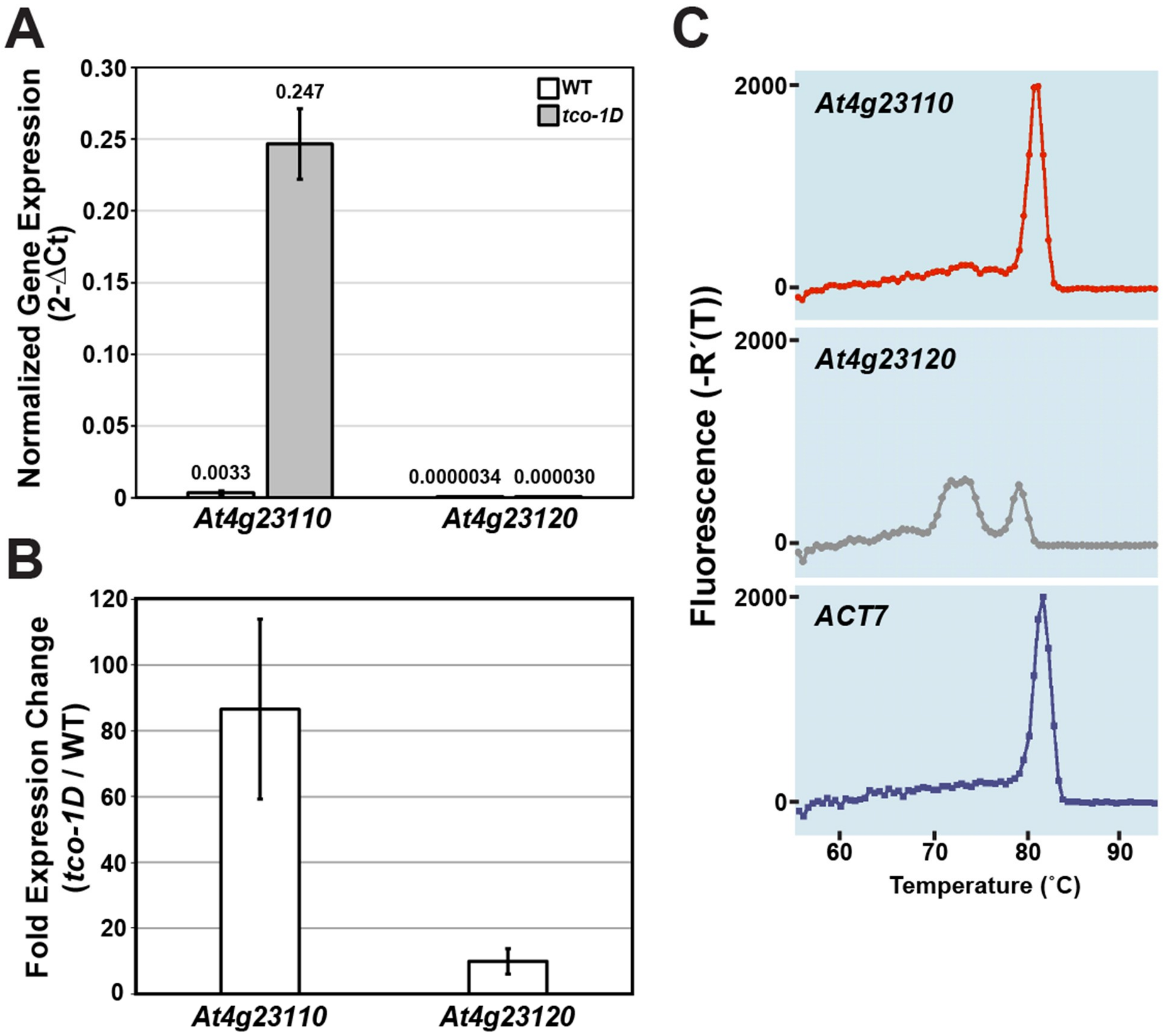

Figure S1. Expression levels of genes flanking the activation-tagging T-DNA in *tco-1D*. (**A**) Quantitative RT-PCR (qRT-PCR) of *At4g23110* and *At4g23120* expression in wild-type (WT) (white bars) and *tco-1D* (grey bars) seedlings at 7 days after germination (DAG). Relative expression of tested genes is normalized against *ACTIN7* ($\Delta Ct = Ct_{TEST} - Ct_{ACTIN7}$). Data are shown as mean +/- SE of two biological replicates. (**B**) qRT-PCR of *At4g23110* and *At4g23120* expression in *tco-1D* seedlings (7 DAG) relative to wild type (WT). Fold expression change (*tco-1D*/WT) was normalized against *ACTIN7* expression. Data are represented as mean +/- SE of two biological replicates. Primer sequences are provided in Table S1. (**C**) Representative dissociation curves of qRT-PCR reactions assessing expression of *At4g23110* (top), *At4g23120* (middle) and *ACT7* (bottom) in *tco-1D* seedlings. Note the strong, sharp peak in each of the *At4g23110* and *ACT7* dissociation curves, indicative of a single, specific PCR product. In contrast, multiple short peaks are apparent in the *At4g23120* dissociation curve, indicative of weak, non-specific amplification. This corrleates with the absence of a detectable *At4g23120* semi-quantitative RT-PCR product in Figure 2B.

A

```
   At4g23110 MGKKSKPFMLSSDAPKPPHRQPYKALLIRAQSSKVNSTDGEATGSQMKLRKQRKAPYKAL 60
    A.lyrata MRNKSQPSRLSPDEASPLPQQPCKALPIRGQSSKVKSRDGEATGSQMKPSKTRKAPYKAL 60
             * :**:*  ** *  .*  :** *** **.*****:* **********  * ********

   At4g23110 LIRARSPGLSEESSSESD-----FSYEADCEASDSSSAESEATNMKPMKNKTARKAPE-- 113
    A.lyrata LIRARSPHSSDEYSSESESSSDEFSYEADGEASDSSSADSDATDVKPMKNKTARKAPISQ 120
             *******  *:* ****:     ****** ********:*:**::************

   At4g23110 ---ARLADKEQNKRRRISEKNNNPEETMSWEPEVKKTL 148
    A.lyrata EPEAGMEIEEPNKRRRISEKDDDSEDTMSWEPEVKKTP 158
                * :  :* *********::: *:***********
```

B

```
   At4g23110 MGKKSKPFMLSSDAPKPPHRQPYKALLIRAQSS---KVNSTDGEATGSQMKLRK---QRK  54
   C.rubella MMRNSQPSTPSNDAPPLPKR-PCKALLIRAQSPEPARVNSAAREATGSKMKPRENTGERR  59
    C.sativa --REAAGSNMTPRRNTGTSRAPYKALLIRAQSPEPARVNSMDREATGSQMTSRRNTGARR 141
               :::     :        * * *********    :***   *****:*. *.    *:

   At4g23110 APYKALLIRARSPGLSEESSSESDFSYEADCEASDSS-----------------------  91
   C.rubella APYKALRMRAQSPEPARVKPKDREATGSKKKSREDSTGTRKAPYKALRMRAQPPEPRFTQ 119
    C.sativa APYKALLIRAQSPEPARVNSTDREATGSQMTARR-NTSTRRAPYKALLIRAQSPEPARVN 200
             ****** :**:**  :. . .: : : .       .:

   At4g23110 --SAESEATNMKPMKNKTARKAPEARLADKE-QNKRRRISEKNNNPEETMSWEPEVKKTL 148
   C.rubella VKPKKTNTARKAP--------TPK--------AGRTIKEQNKEDAREGTMSAEPEAKETV 163
    C.sativa STDREAAGSTMESRRNTGTSRVPYKALLIRARSPEPTISADSEDAREETMSAEP------ 254
                 ::  :            .*           .     :.::  * *** **

   At4g23110 -------- 148
   C.rubella AKKNHKQD 171
    C.sativa -------- 254
```

C

```
   At4g23110 MGKKSKPFMLSSDAPKPPHRQPYKALLIRAQSSKV---NSTDGEATGSQMK------LRK 51
E.salsuginem MSDNSQLRGRLPHVASPHPKRPYKSLRIRAQSPEPVEENSMDNEATGSTEKGMKPRNDNT 60
             *..:*:      .. .*  ::***:* ***** :    ** *.*****  *       ..

   At4g23110 QRKAPYKALLIRARSPGL-SEESSSESDFSYEADCEASDSSSAESEATNMKPMKNKTARK 110
E.salsuginem SRKVPYKALLIRARSPEPYHEENSTDSEATSSQESE-------------VKPRKNNGAKK 107
             .**.************    **.*::*: : . :.*             :** **: *:*

   At4g23110 APEARLADKEQNKRRRISEKNNNPEETMSWEPEVKKTL 148
E.salsuginem DLKNRLE----------TRKNNTPKRIVSHEPELRR-- 133
               : **           :.***.*:. :* ***:::
```

D

```
   At4g23110 MGKKSKPFMLSSDAPKPPHRQPYKALLIRAQSSKVNSTDGEATGSQMKLRKQRKAPYKAL 60
   R.sativus ---------M-SDKFKPPRK----TLPRR---SLLPEPSGESSTDDEANEVERNVPYKAL 43
     B.napus PNRISTPRSM-SDKYKLPRK----TLRRR---SLSPELSGESSTDDEANKVERKVPYKAL 69
                      : **  * *::    :*  *   *   . .**:: .:   . :*:.*****

   At4g23110 LIRARSPGLSEESSSESDFSYEADCEASDSSSAESEATNMKPMKNKTARKAPEARLADKE 120
   R.sativus LMRARPF---DEDDS-------TDSEATDSDATSPEELEESPKKNNDAIDEP--------  85
     B.napus LIRPRPFEPFDEDDSTASD--ATDSDATDSDVTSPEGLKVSPKKSNDSINEP-------- 119
             *:* *     :*..*       :*.:*:**. :. *  : .* *.: : . *

   At4g23110 QNKRRRIS-EKNNNPEETMSWEPEVKKTL------------------------------- 148
   R.sativus SNKTRRLHRRKMNAPRRIISPEAEVNQTDAKPCNSGGSGKAHSDQRVAPEEP-------- 137
     B.napus SNKKRRLHRKKMYTPRKFISPEAEVNQTDANPCNSGRSEKALSDQRVSSTESKDARDLFY 179
             .** **:  .*   *.. :* * **::*

   At4g23110 ------------------------------------------------------       148
   R.sativus ------------------------------------------------------       137
     B.napus SFYYDEVFADSVKHSCIVHFVPENKQIPNRKEHPGLIVQKVYNIALEEAVEDHS       233
```

Figure S2. *Arabidopsis* TCO/At4g23110 shares sequence similarity to proteins found in other *Brassicaceae* species. Amino acid sequence alignments of *Arabidopsis* TCO/At4g23110 and related proteins from *Arabidopsis lyrata* (**A**), *Capsella rubella* and *Camelina sativa* (**B**), *Eutrema salsugineum* (**C**), and *Raphanus satvius* and *Brassica napus* (**D**). Results are based on sequence alignments generated by the program *Clustal Omega* [68]. Amino acid residues of the same color share chemical properties. Amino acid positions marked with an asterisk (*) are fully conserved, while those marked with a colon (:) and period (.) have properties that are strongly and weakly similar, respectively. Genbank (National Center for Biotechnology Information (NCBI)) locus identifiers for TCO-like proteins are XP_002869789 (*A. lyrata*), XP_006285633 (*C. rubella*), XP_010448771 (*C. sativa*), XP_024016111 (*E. salsugineum*), XP_018479164 (*R. sativus*), and CDY10916 (*B. napus*).

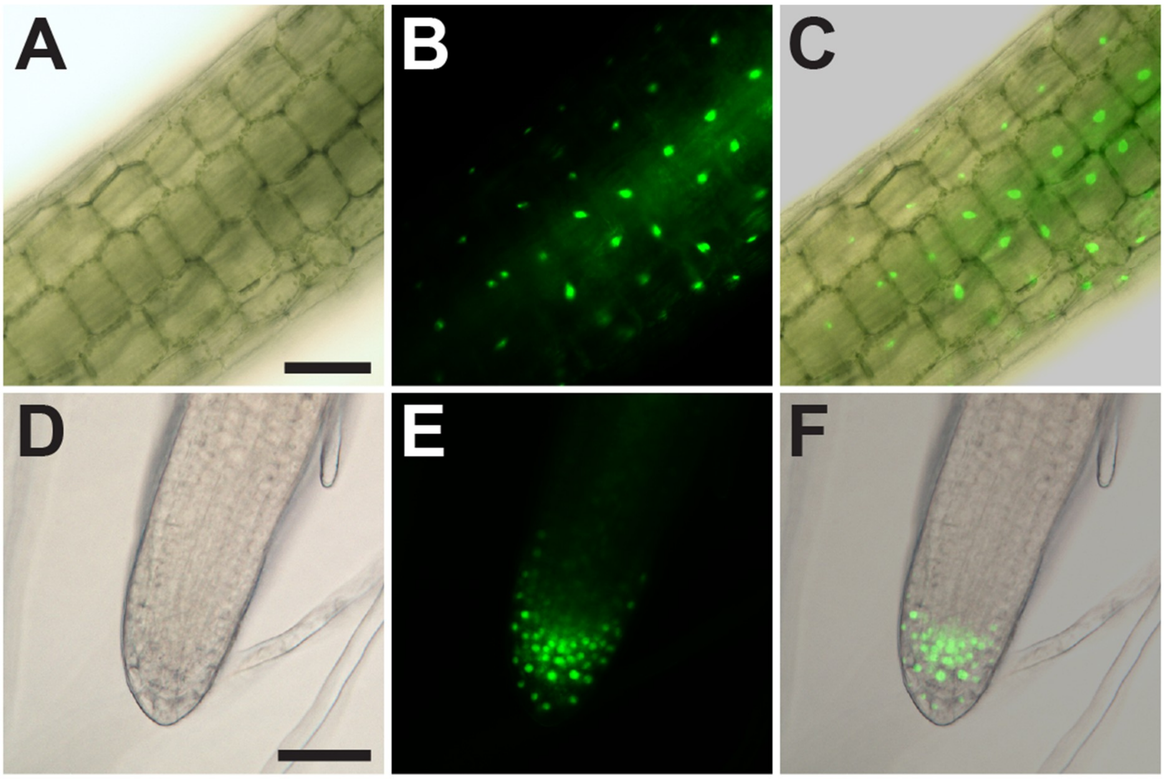

Figure S3. Localization of TCO-GFP in stable transgenic *Arabidopsis* lines. (**A**-**F**) The hypocoytl (**A**-**C**) and root tip (**D**-**F**) of stable 2x35Sp::TCO-GFP transformants are depicted. Brightfield (**A**,**D**), GFP (**B**,**E**) and merged images (**C**,**F**) show punctate subcellular accumulation patterns of TCO-GFP that are consistent with nuclear localization. Bars: (**A**-**C**) 1 mm; (**D**-**F**) 0.5 mm.

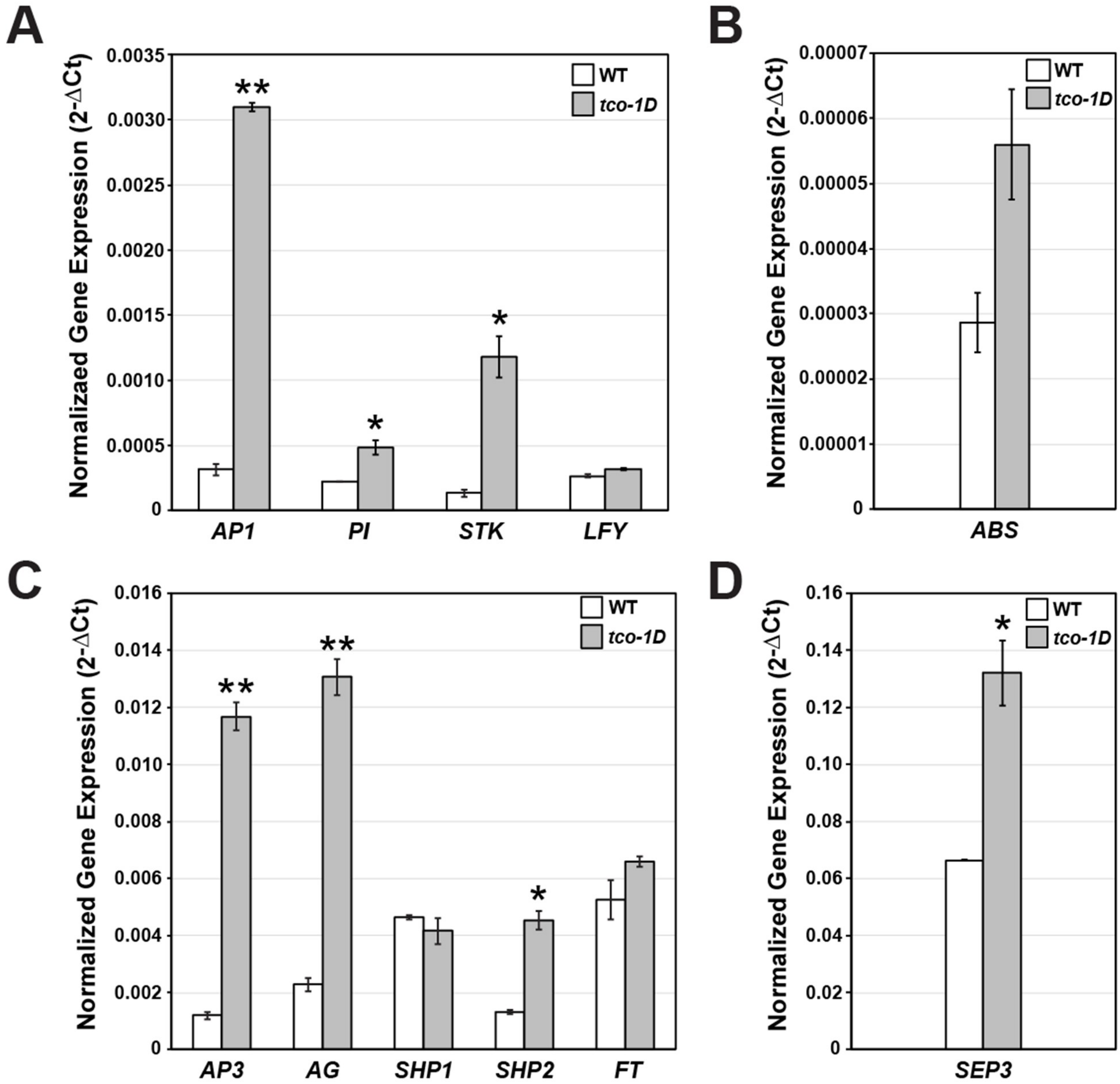

Figure S4. Expression levels of floral genes in wild-type and *tco-1D* vegetative tissues. Quantitative RT-PCR of *AP1*, *PI*, *STK*, *LFY* (**A**), *ABS* (**B**), *AP3*, *AG*, *SHP1*, *SHP2*, *FT* (**C**) and *SEP3* (**D**) expression in 7 days after germination wild-type (WT) (white bars) and *tco-1D* (grey bars) seedlings. Relative expression of tested genes is normalized against *ACT7* ($\Delta Ct = Ct_{TEST} - Ct_{ACT7}$). Data are shown as mean +/- SE of two biological replicates. Statistically significant differences between expression levels in *tco-1D* relative to WT are indicated (* $p < 0.05$, ** $p < 0.005$; two-tailed *t*-test). These graphs are alternative representations of the same data presented in Figure 3F, with relative expression levels of each gene in wild-type and *tco-1D* seedlings displayed separately. Primer sequences are provided in Table S1.

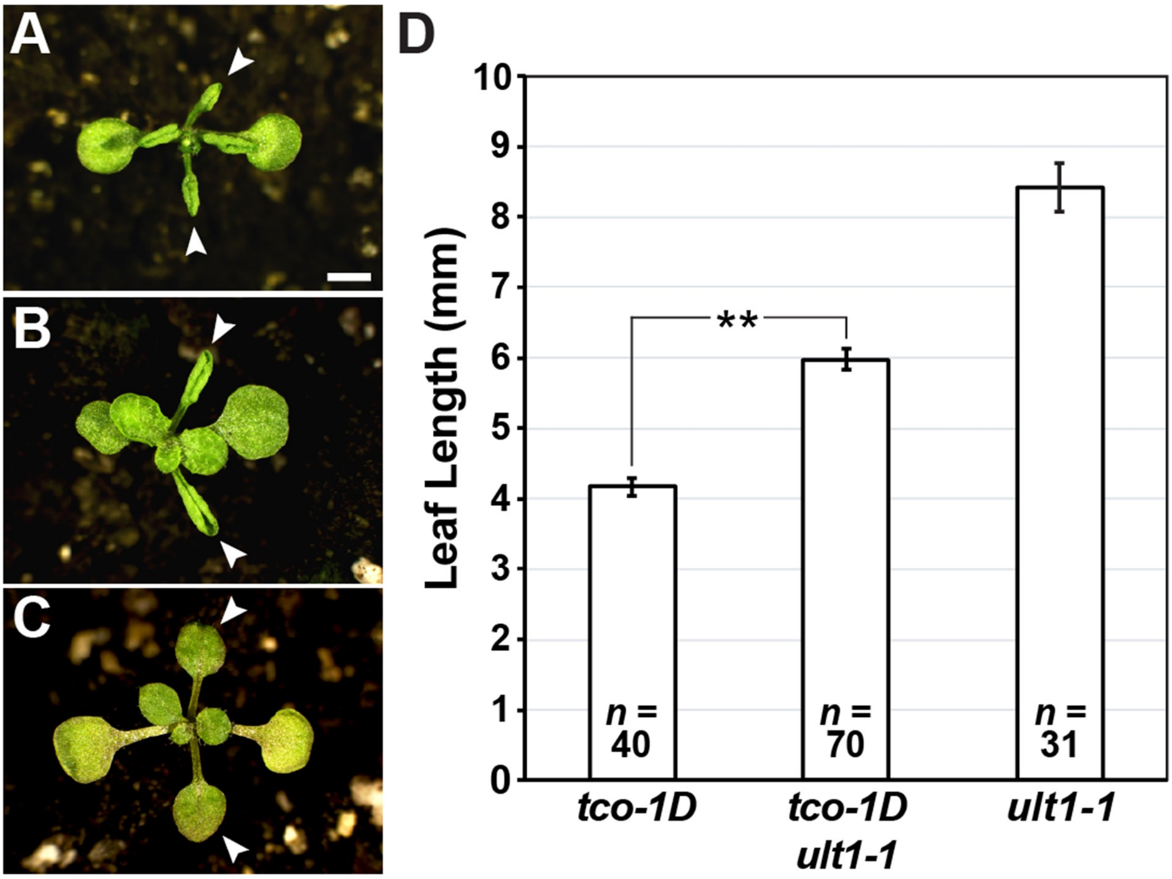

Figure S5. Developmental defects of *tco-1D* leaves are suppressed by the semi-dominant mutant *ult1-1*. (**A**-**C**) Apical views of 17 days after germination *tco-1D* (**A**), *tco-1D ult1-1* (**B**) and *ult1-1* (**C**) plants, with first vegetative leaves denoted (arrowheads). Bars: 2 mm. (**D**) Lengths of first vegetative leaves of *tco-1D*, *tco-1D ult1-1* and *ult1-1* are shown. Data are represented as mean +/- SE. Asterisks denote statistically significant difference (** $p < 0.001$; two-tailed t-test). Sample size (*n*) of each genotype is provided.

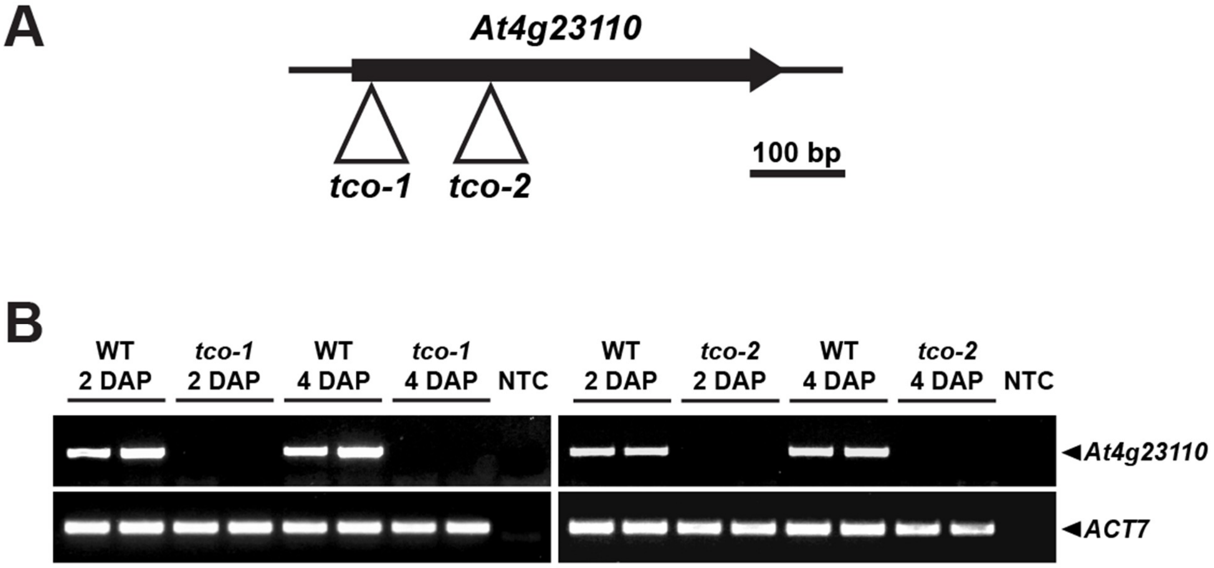

Figure S6. *TCO* expression in the *Arabidopsis* T-DNA insertion alleles *tco-1* and *tco-2*. (**A**) Schematic of *TCO*/*At4g23110* (black arrow) showing positions of T-DNA insertions in *tco-1* (SALK_018803) and *tco-2* (SALK_112041). (**B**) Semi-quantitative RT-PCR testing expression of *TCO*/*At4g23110* in *tco-1* (left) and *tco-2* (right) siliques (2 and 4 days after pollination (DAP)) relative to wild type (WT). Two biological replicates are depicted. Expression of *ACTIN7* (*ACT7*) serves as an internal control and no-template controls (NTC) are included. RT-PCR primers span the T-DNA insertion sites of *tco-1* and *tco-2*. Primer sequences are provided in Table S1.

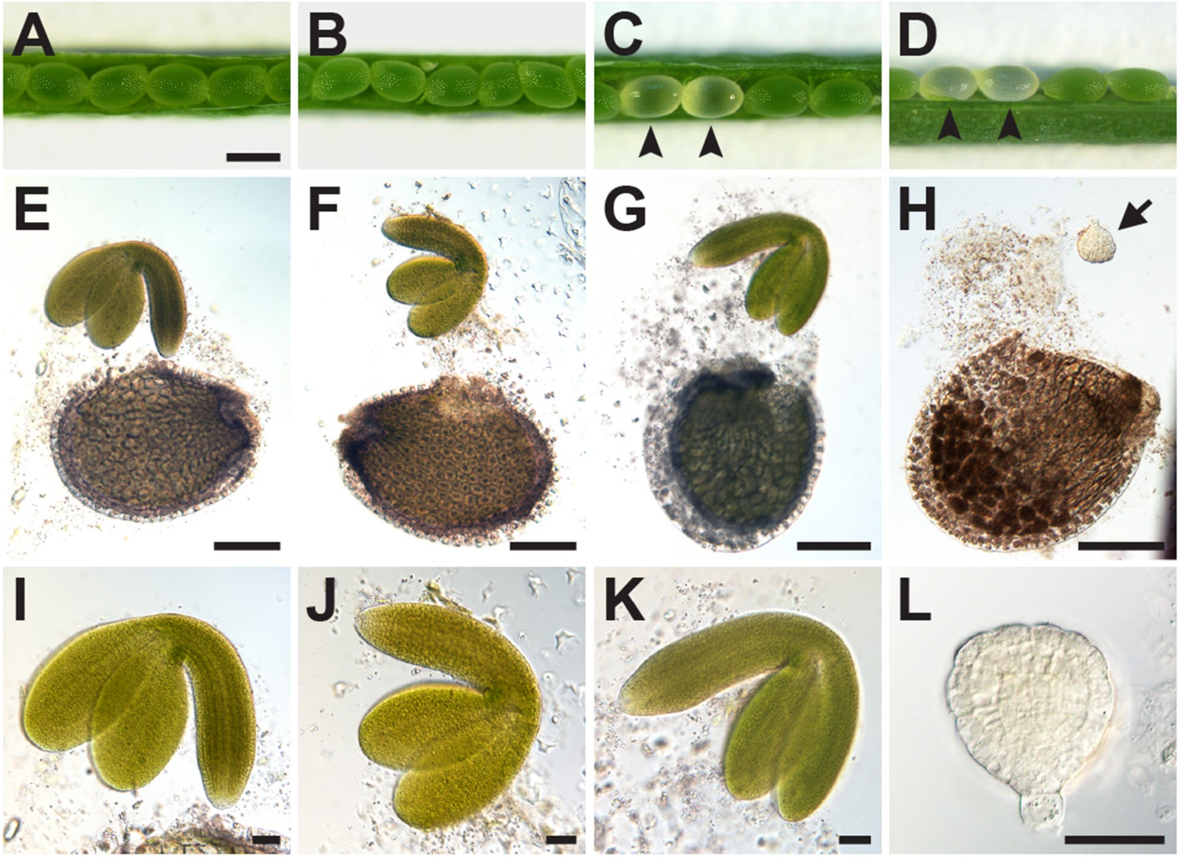

Figure S7. Seed defects of *Arabidopsis* insertion mutant *tco-1*. (**A**-**C**) Top view of *Arabidopsis* seeds at 9 days after pollination (DAP) in wild-type (**A**), *tco-2* (**B**), and *tco-1* (**C**) siliques. Affected *tco-1* seeds are white-to-light green in appearance (arrowheads). (**D**) Side view of *tco-1* seeds depicted in (**C**). (**E**-**L**) Embryos dissected from fixed and partially cleared seeds at 9 DAP. Wild-type (**E**,**I**), *tco-2* (**F**,**J**) and *tco-1* embryos from green seeds (**G**,**K**) are at the bending cotyledon stage. (**H**,**L**) White-to-light green *tco-1* seeds harbor embryos at the globular stage (arrow in **H**). Bars: (**A**-**D**) 0.5 mm; (**E**-**H**) 200 μm; (**I**-**L**) 50 μm.

Table S1. List of primers used in this study.

**Name**	**Sequence (5'-to-3')**
AP1 qRTPCR F	CGACGTCAATACAAACTGGTCGAT
AP1 qRTPCR R	CTTTAGGGCTCATTGCTTGCA
AP3 qRTPCR F	TGAGCTGGAACTAAGAGCTGAAG
AP3 qRTPCR R	GTTGGGGTAATAGTGGTGATGGT
PI qRTPCR F	ACTTGAAAAATCTGATGGCTGTCG
PI qRTPCR R	TTGCTATAGCCATCTCCTGTTGTT
AG qRTPCR F	AACGCAATCTCAACCGTTTGATT
AG qRTPCR R	CTTACACTAACTGGAGAGCGGTT
ABS qRTPCR F	AGATAACAACAACATGTACCGTTGG
ABS qRTPCR R	CCTGGTTTATAGCACTGAAGCTGC
SHP1 qRTPCR F	AAGGACGTCTTGAAAAAGGAATC
SHP1 qRTPCR R	ACTGTCGTCCCTTGTATCACACT
SHP2 qRTPCR F	AAGAAGCACGAGATGTTAGTTGC
SHP2 qRTPCR R	GACTCGTAAACTGTCCCTTGATG
STK qRTPCR F	TGGTTCTGGATCTGGTAATGG
STK qRTPCR R	CAGAGAGTTATTGCAGCTCGG
SEP3 qRTPCR F	AAGAAGAGGTTGATCACTACGGTC
SEP3 qRTPCR R	ATACCCGATCTGAAGAATGGGTTC
LFY qRTPCR F	CCCACCAAGGTGACGAACCA
LFY qRTPCR R	ACAGTGAACGTAGTGTCGCATT
FT qRTPCR F	GAACAACCTTTGGCAATGAGATT
FT qRTPCR R	CACCCTGGTGCATACACTGTT
ACT7 RTPCR F	GGTGAGGATATTCAGCCACTTGTCTG
ACT7 RTPCR R	ACCATGACACCAGTGTGCCT
At4g23110 RTPCR F	AGCAACTGGTTCGCAAATGAAGC
At4g23110 RTPCR R	GTTCTTCATCGGCTTCATATTGG
At4g23120 RTPCR F	GCTGGTGATTATGATTCTTTCTGGC
At4g23120 RTPCR R	CATCATCCTTCAATATTGGTTCCTC
TCO prom F GUS reporter	GATACTCCTATGAATTATGAAGCTGTGC
TCO gene F GFP fusion	GAATTCTTTCCTTTGATCTTTCAGG
TCO gene R GUS/GFP	AAGCTTAGAGTCTTTTTAACTTCTGGTTC
TCO 3'UTR F GUS reporter	TCTAGAAAGAAGATTTCGACAGAAGAAGC
TCO 3'UTR R GUS reporter	ACTAGTTAGTAAGTCGTCTCGGCAGTTA
tco spans insertion F	TTCAGGTTTGCAAGTTCCAAATGGG
tco spans insertion R	GTCTTTTTAACTTCTGGTTCCCAGG
TCO Y2H F	GTCGACAATGGGGAAAAAATCTAAGCC
TCO Y2H R	GCGGCCGCTCAAAGAGTCTTTTTAACTTC
AtCKA1 Y2H F	AGCGTCGACAATGATAGATACGCTTTTCTTC
AtCKA1 Y2H R	GAGCGGCCGCTGTGTTTCATTGACTTCTCATTC
AtCKA2 Y2H F	GAAGTCGACCATGCACCTAATCTTCTTCTTCTCC
AtCKA2 Y2H R	AAGCGGCCGCGACATCTCTATTGAGTCCTCATTC
AtCKA3 Y2H F	ATCGTCGACGATGTCGAAAGCTAGGGTTTATACAG
AtCKA3 Y2H R	CAAGCGGCCGCTTTACTGAGTTCGTAGTCTGCTGC
TCO SDM S75A F	GTGAGGAAAGCTCTGCGGAGAGTGACTTTTCC
TCO SDM S75A R	GGAAAAGTCACTCTCCGCAGAGCTTTCCTCAC
TCO SDM S75D F	GTGAGGAAAGCTCTGATGAGAGTGACTTTTCC
TCO SDM S75D R	GGAAAAGTCACTCTCATCAGAGCTTTCCTCAC
TCO pGEX F	TTGCAATCTAGAAATGGGGAAAAAATCTAAGCCG
TCO pGEX R	TAGAGGGTCGACTCAAAGAGTCTTTTTAACTTCTG
